# Identifying Risk of Viral Failure in Treated HIV-Infected Patients Using Different Measures of Adherence: The Antiretroviral Therapy Cohort Collaboration

**DOI:** 10.3390/jcm7100328

**Published:** 2018-10-05

**Authors:** Suzanne M. Ingle, Heidi M. Crane, Tracy R. Glass, Benita Yip, Viviane D. Lima, M John Gill, Nikola Hanhoff, Adriana Ammassari, Michael J. Mugavero, Jan P. Tate, Jodie Guest, Nicholas L. Turner, Margaret T. May, Jonathan A. C. Sterne

**Affiliations:** 1 Population Health Sciences, Bristol Medical School, University of Bristol, Bristol BS8 2PS, UK; nicholas.turner@bristol.ac.uk (N.L.T.); margaret.may@bristol.ac.uk (M.T.M.); jonathan.sterne@bristol.ac.uk (J.A.C.S.); 2 Clinical Epidemiology and Health Services Research Core, Center for AIDS Research, University of Washington, WA 98104, USA; hcrane@uw.edu; 3 Swiss Tropical and Public Health Institute, CH-4002 Basel, Switzerland; tracy.glass@swisstph.ch; 4 British Columbia Centre for Excellence in HIV/AIDS, Vancouver, BC V6Z 1Y6, Canada; byip@cfenet.ubc.ca (B.Y.); vlima@cfenet.ubc.ca (V.D.L.); 5 Division of Infectious Diseases, University of Calgary, Calgary, AB T2N 4N1, Canada; john.gill@albertahealthservices.ca; 6 Southern Alberta Clinic, Calgary, AB T2R 0X7, Canada; nikola@hanhoff.com; 7 Istituto Nazionale Malattie Infettive “L. Spallanzani”, IRCCS, 00149 Rome, Italy; adriana.ammassari@inmi.it; 8 Division of Infectious Disease, Department of Medicine, University of Alabama, Birmingham, AL 35294, USA; mmugavero@uab.edu; 9 Yale University School of Medicine, West Haven, CT 06510, USA; Janet.Tate2@va.gov; 10 HIV Atlanta VA Cohort Study (HAVACS), Rollins School of Public Health at Emory University, Atlanta, GA 30322, USA; jodie.guest@emory.edu

**Keywords:** HIV, antiretroviral therapy, adherence, viral failure, cohort studies

## Abstract

Adherence to antiretroviral therapy (ART) is critical for successful treatment of Human Immunodeficiency Virus (HIV), but comparisons across settings are difficult because adherence is measured in different ways. We examined utility of different adherence measures for identification of patients at risk of viral failure (VF). Eight cohorts in the ART Cohort Collaboration contributed data from pharmacy refills or self-report questionnaires collected between 1996 and 2013 (*N* = 11689). For pharmacy data (*N* = 7156), we examined associations of percentage adherence during the 1st year of ART with VF (>500 copies/mL) at 1 year. For self-report data (*N* = 4533), we examined 28-day adherence with VF based on closest viral load measure within 6 months after questionnaire date. Since adherence differed markedly by measurement type, we defined different cut-off points for pharmacy (lower <45%, medium 45–99%, higher 100%) and self-report (lower ≤95%, medium 96–99%, higher 100%) data. Adjusted odds ratios (ORs) for VF in lower and medium, compared to higher adherence groups, were 23.04 (95% CI: 18.44–28.78) and 3.84 (3.36–4.39) for pharmacy data. For self-report data, they were 3.19 (2.31–4.40) and 1.08 (0.80–1.46). Both types of measure were strongly associated with VF. Although adherence measurements over longer time-frames are preferable for prediction, they are less useful for intervention.

## 1. Introduction

Adherence to antiretroviral therapy (ART) sufficient to suppress viral replication is critical for successful treatment of HIV [1,2,3,4,5]. Traditionally, it has been suggested that patients should be at least 95% adherent to optimise virologic outcomes [6]. However, this threshold may not apply to new ART regimens requiring less frequent dosing. Better-defining the relationship between adherence and viral suppression could enable early identification of patients with problematic levels of adherence and, hence, facilitate proactive interventions to prevent treatment failure. 

Methods to assess medication adherence include self-report questionnaires, pharmacy-refill data, pill counts, electronic drug monitoring, and monitoring drug levels in the blood [7,8]. However, research findings can depend on which of these methods is used. The most commonly used adherence measures are based on pharmacy-refill data or self-report questionnaires: their advantages and disadvantages have been discussed extensively. Adherence derived using self-report questionnaires may be overestimated because of the tendency for patients to provide perceived desirable answers [9], but self-report provides an easy and fast way to measure adherence. Adherence estimated from pharmacy-refill data is likely to be more objective and accurate than self-report, but requires protocols for data collection and management [10]. Different measurement and calculation methods used on pharmacy data can lead to inconsistencies [11,12] when data are compared across cohorts or used in pooled analyses.

We examined whether different adherence measures can be calibrated for use in pooled analyses by using different cut-off points. In an era of large international HIV cohort collaborations, the appropriate use of data combined from different settings is ever more important. Identifying those at risk of viral failure is crucial for successful treatment, and so we also investigated the utility of different adherence measures for identifying those at risk of viral failure, among cohorts participating in the ART Cohort Collaboration (ART-CC).

## 2. Methods

### 2.1. Cohorts and Patients

ART-CC is a collaboration of cohort studies from Europe and North America established with the aim of describing the prognosis of antiretroviral-naïve patients starting ART [13] (www.bris.ac.uk/art-cc). Prospective cohort studies were eligible if they enrolled at least 100 HIV-1-positive ART-naïve patients aged ≥16 years who started ART on at least three drugs, including nucleoside reverse-transcriptase inhibitors (NRTIs), protease inhibitors (PIs), or non-nucleoside reverse-transcriptase inhibitors (NNRTIs), with a median follow-up of at least 1 year. All cohorts use quality control procedures and provided a predefined and anonymised set of demographic, laboratory, and clinical variables. 

Eight cohorts, whose patients were enrolled in differing healthcare systems (see Table 1), contributed adherence data, which were collected between 1996 and 2013: Italian Cohort of Antiretroviral-Naïve Patients (ICONA) [14], the Swiss HIV Cohort Study (SHCS) [15], the HAART Observational Medical Evaluation and Research (HOMER) [16] and the Southern Alberta Clinic Cohort (Alberta) (Canada), the 1917 Clinic Cohort University of Alabama (UAB), the University of Washington HIV Cohort (UW), the Veterans Affairs Cohort Study (VACS), and the HIV Atlanta Veterans Affairs Cohort Study (HAVACS) (USA). The database was updated in September 2013. Institutional review boards approved data collection at all sites, and each individual cohort has their own separate approvals. Data from ICONA and SHCS were pooled in September 2013 within COHERE in EuroCoord (www.cohere.org and www.EuroCoord.net). The NHS Health Research Authority South West—Cornwall and Plymouth Research Ethics Committee, UK, has approved the ART-CC study (REC reference 12/SW/0253). 

### 2.2. Deriving Measures of Adherence and Viral Suppression

Four cohorts provided adherence data from pharmacy refills (Alberta, HOMER, VACS, and HAVACS) and four from self-report questionnaires (ICONA, UAB, Washington, and SHCS). Only the VACS cohort provided data from both pharmacy refills and self-report questionnaires. Adherence data were available for only a subset of the patients in ICONA, SHCS, UAB, UW, and HAVACS as either, not all patients consented, or they spoke a different language or answered a different version of the adherence question, which was not comparable as there was too little discrimination (for example, one of the questions asked was “When was the last time you missed any of your antiretrovirals?” Yesterday, Within the past week, 1–2 weeks ago, 3–4 weeks ago, Never skip medications). However, the choice of these subsets should not have introduced bias as patient characteristics in the analysis sample were similar to those of included patients from the same cohort. Adherence data were available for all patients in Alberta, HOMER, and VACS.

**Figure 1:**
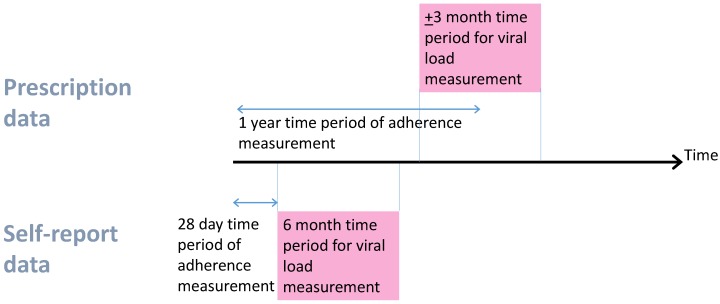
Data timelines. For prescription measures, there was a median of −1 day between adherence and viral load measure and, for self-report, a median of 84 days.

Self-report questionnaires asked about behaviour in the last 28 days, and were administered when patients attended clinical visits. For ICONA, UAB, and UW, patients were asked how often they took their antiretrovirals in the last 28 days (month for ICONA), and asked to mark it on a visual analogue scale ranging from 0 (never) to 100 (always). We used this as the percentage adherent. For SHCS, the percentage adherence over the last 4 weeks was calculated based on patient-reported number of missed doses. Questionnaires were administered multiple times, but at infrequent intervals. In SHCS, the median time between questionnaires was 5 months, UW 7.5 months, ICONA 6.5 months, UAB 6 months. Viral suppression (≤500 copies/mL) was based on the closest viral load measure within 6 months after the questionnaire date (Figure 1).

Pharmacy refill data record the date that a prescription was filled and how many days’ worth of drug was supplied. From this, we know when a patient should return for another refill. For pharmacy data, we derived 1-year percentage adherence and corresponding viral suppression (≤500 copies/mL) based on the viral load closest to (and within ± 3 months of) the 1-year time point (Figure 1). Percentage adherence in the first year was derived as the number of days’ worth of drug supplied, divided by the number of days on which the patient could have taken drugs. For most patients, the denominator was 365 days, but it was less if they died, moved out of the area, or were advised to interrupt treatment. Patients were only included if they had the potential for at least 1 year of pharmacy data (i.e., patients with only 6 months of data before the database close date were not included). The method for calculating the numerator for this ratio differed between cohorts. In HOMER, if a patient obtained their next prescription before their current one had run out, then this extra days’ supply was not included in the numerator. If any individual drug was stopped and started again with a gap of 30 days or less, then this was assumed to be a continuous interval of drug supply because patients could be using up those extra days’ supply (stockpiled medications). In Alberta, VACS, and HAVACS, if a patient returns early for their refill, then the days covered for that refill do not start until the first prescription has run out. This is described by the algorithm reported by Steiner et al. [12].

### 2.3. Analysis

Patients were only included in the analysis if they had data on both adherence and viral load. Median levels of adherence were compared between patients who were and were not suppressed. We fitted receiver operating characteristic (ROC) curves (plots of (1-specificity) against sensitivity) to assess the relationship between percentage adherence and viral suppression, separately in each cohort. The area under the ROC curve (AUROC) was used to measure the discrimination of the adherence measure for viral suppression: an AUROC of 0.5 corresponds to discrimination no better than expected by chance, and 1 to perfect discrimination.

Logistic regression was used to examine associations of percentage adherence with viral suppression. As a first step, adherence was categorised as <95% and ≥95%, because this is a widely used classification. However, for self-report data, this categorisation had little meaning as most patients rated their adherence to be very high. After examining the distribution of adherence among cohorts with self-report and pharmacy data, we categorised adherence differently for cohorts with pharmacy data (as <45% (low adherence), 45–99% (medium), and 100% (high)) and for cohorts with self-report data (as ≤95% (low adherence), 96–99% (medium), and 100% (high)). These cut-offs were chosen to make proportions as similar as possible across the pharmacy and self-report data. We fitted crude and adjusted (for cohort, age, gender, CD4, and viral load at ART start, AIDS at ART start, and transmission risk group) logistic regression models to assess the association of adherence with viral suppression. Stata^TM^ version 13.0 [17] was used for all analyses.

### 2.4. Sensitivity Analyses

We considered whether the effect of adherence on suppression was modified by age, and conducted sensitivity analyses (1) using a viral load cut-off of 50 copies/mL to define viral suppression; (2) restricted to the most recent 5 years of data (2009–2013) in order to report on regimens which are currently prescribed; and (3) restricted to patients who were still on their baseline ART regimen at the time of the outcome measure. The different time frames used for pharmacy and self-report adherence measures led us to consider using different time frames (3 and 6 months) for pharmacy data, which could be more easily compared with self-report.

There has been discussion in the field of adherence to diabetes medication that patients who are “over-adherent” (those who stockpile their medications and so appear to have >100% adherence) may be different from the rest of the population [18,19]. We were able to assess whether patients from the Alberta, VACS, and HAVACS cohorts were over-adherent, and ran a sensitivity analysis excluding those patients. 

The VACS cohort had both pharmacy and self-report measures available for direct comparison between the two methods. As the pharmacy measure is based on a 12-month period, we used the question “In the past 12 months, when you take your HIV medications, how often do you take all the medications you’re supposed to?” from self-report questionnaires for comparison (possible answers: never, some, about half, most of the time, all of the time). In their self-report data, VACS did not ask a question using the visual analogue scale, so we were unable to calculate percentage adherence.

## 3. Results

Adherence data were available on 11,689 patients: 7156 from the four cohorts with pharmacy data, and 4533 from the four cohorts with self-report data. Table 1 shows characteristics of patients at the time of ART initiation, overall and by cohort. The majority were male (83.9%) and the median age was 41 years. Median CD4 count and viral load at ART start were 200 cells/mm^3^ and 4.9 log_10_ copies/mL, respectively. VACS and HAVACS are cohorts of military veterans, most of whom are men and were older, on average. The distribution of risk groups for HIV acquisition also differs between cohorts: VACS has poorly recorded transmission risk in their cohort and HOMER has a high proportion of injection drug users (IDU) and patients with unknown transmission risk. Three out of eight cohorts supplied refill drugs automatically without the patient requesting it.

**Figure 2:**
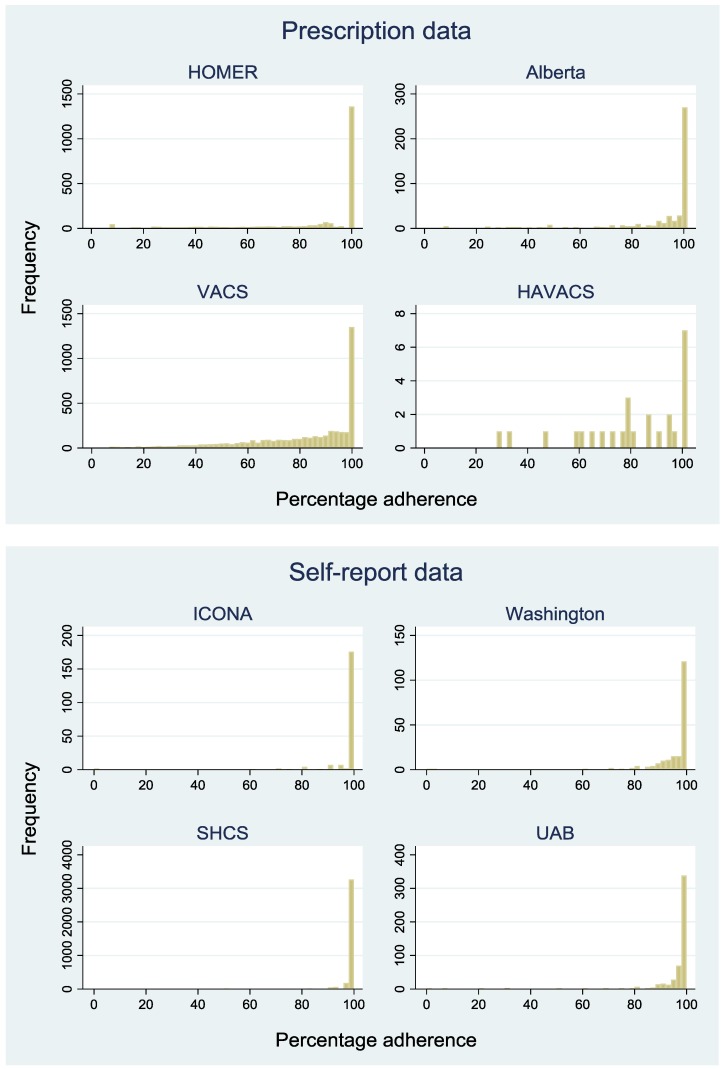
Distribution of percentage adherence in each cohort.

Figure 2 shows the distribution of adherence across the eight cohorts. In cohorts with self-report data, most patients reported high levels of adherence in the previous 28 days (median 100%, interquartile range (IQR): 100–100). In cohorts with pharmacy data, estimated adherence was much more variable (median 94%, IQR: 72–100). In cohorts with self-report data, adherence takes a limited number of values as patients are asked to mark adherence on a visual scale, rather than report a number. 

Table 2 shows levels of adherence by cohort, together with their associations with viral failure. In cohorts with self-report data, 8.6% of patients had viral failure, compared with 31.9% in cohorts with pharmacy data. Estimated levels of adherence were much greater for cohorts with self-report than pharmacy refill data. For cohorts with pharmacy data, 9% of patients were in the “low” category, 49% in “medium” and 42% in “high”. For cohorts with self-report data, 8% of patients were in the “low” category, 17% in “medium” and 75% in “high”. 

Among cohorts with pharmacy data, estimated adherence was much lower in patients with subsequent viral failure than in those who were suppressed. This pattern was not seen to the same extent among cohorts with self-report data. Associations of adherence with viral failure were similar for Alberta and HOMER, but weaker for VACS. For HAVACS, there was little evidence that adherence was associated with viral failure, although associations were imprecisely estimated because the sample size was small. Using adherence data pooled over all cohorts with pharmacy data, the odds ratios (ORs) (95% CI) for viral failure in lower and medium, compared to higher adherence groups, were 26.1 (21.1–32.4) and 4.4 (3.9–5.0), respectively. After adjusting for baseline covariates, the ORs were 23.0 (18.4–28.8) and 3.8 (3.4–4.4), respectively. We also compared lower with medium levels of adherence. Across all cohorts, there was strong evidence that lower adherence led to higher rates of viral failure compared to medium adherence.

Among cohorts with self-report data, there was little evidence that medium level adherence was associated with higher odds of viral failure. However, self-reported low adherence was associated with higher odds of viral failure. Using adherence data pooled over all cohorts with self-report data, the ORs (95% CI) for viral failure in lower and medium, compared to higher adherence groups, were 3.1 (2.4–4.2) and 1.2 (0.9–1.5), respectively. After adjusting for baseline covariates, the ORs were 3.2 (2.3–4.4) and 1.1 (0.8–1.5), respectively. As with pharmacy data, we also compared lower with medium levels of adherence and, again, found that lower levels of adherence led to higher rates of viral failure compared to medium adherence.

**Figure 3:**
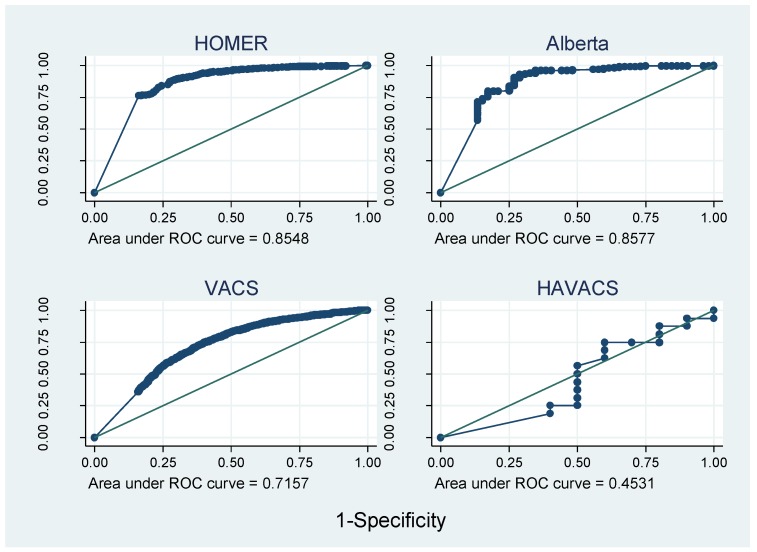
Receiver operating characteristic (ROC) curves for cohorts with pharmacy data.

**Figure 4:**
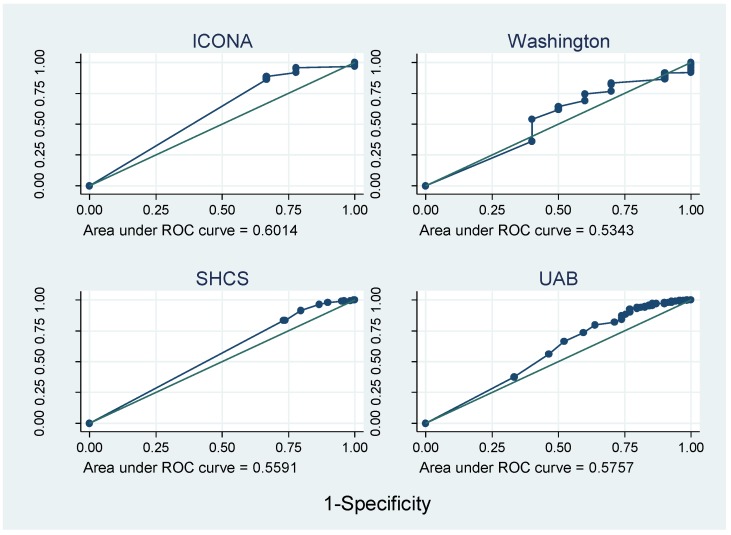
ROC curves for cohorts with self-report data.

Figure 3 and Figure 4 show that the AUROC varied greatly between cohorts, from 0.45 in HAVACS to 0.86 in Alberta. Overall, compared with self-report, pharmacy adherence data better discriminated those patients with viral failure as they have a larger range of continuous adherence values, and hence more points on the ROC curve. AUROCs were relatively consistent between cohorts using the same type of adherence data, except there was no evidence that adherence predicted viral failure in the HAVACS cohort.

### Sensitivity Analyses

For cohorts with pharmacy data, there was weak evidence that age modified the effect of adherence on failure (Web Table 1, see Supplementary Materials). Among patients aged ≥50 years, there were less pronounced differences in the magnitudes of associations of low and medium levels of adherence with viral failure than for younger patients. In sensitivity analyses defining viral failure as >50 copies/mL, results were similar to the main analysis, but attenuated (Web Table 2).

When restricting to the most recent 5 years of data (2009–2013), data were only available from the Alberta, ICONA, SHCS, UAB, and Washington cohorts. Most were cohorts with self-report data and odds ratios were similar to those for cohorts with self-report data in the main analyses (Web Table 3). Results of analyses restricted to those remaining on their first ART regimen at the time of the outcome were similar to the main analyses (Web Table 4). We also considered whether restricting pharmacy data to just 28 days of data (and 3 or 6 months) made results more similar to those from self-reported adherence. As most prescriptions are filled for at least 28 days, there was little variability in adherence when considering the data this way. When comparing pharmacy data over 3 and 6 months, the AUROCs improved as the time increased (Web Table 5). We assessed over-adherence in the Alberta, VACS, and HAVACS cohorts, and found that results were similar after excluding over-adherent patients.

To compare self-report and pharmacy measures directly, we used data from 348 VACS patients. Due to the way the self-report question was asked (see Methods section), we could not calculate adherence percentage, and so had to use the raw data. As self-reported adherence increased, so did the pharmacy adherence measure (Web Table 6, test for trend *p* < 0.001). We compared the associations of pharmacy/self-report adherence measures with viral failure in 331 patients. Web Table 7 shows AUROCs for both measures of adherence with viral failure were similar.

## 4. Discussion

### 4.1. Main Results

We combined data from eight HIV cohort studies: four with adherence measured using patient self-report and four using pharmacy refill data. There was heterogeneity between cohorts in both levels of adherence and viral failure. Both self-report and pharmacy refill adherence data can be used to predict viral failure and show evidence that increasing adherence decreases the risk of viral failure. Pharmacy measures were much more strongly associated with viral failure than self-reported measures, although this may be due, in part, to the longer time-frame used in the pharmacy data and the higher % with viral failure in the pharmacy cohorts. When considering data from the VACS cohort, which provided pharmacy and self-report measures, the strength of associations of the different measures with viral failure were similar. To make full use of adherence data, there needs to be an awareness of cohort-specific factors affecting adherence. For example, in the Alberta cohort, patients with very poor adherence may have drugs stopped pending correction of underlying issues compromising adherence.

### 4.2. Context

Pharmacy refill and self-report are different tools for measuring adherence that have different characteristics and uses [20]. Short-term self-reported adherence may provide a better snapshot of recent adherence behaviour, which may be beneficial in clinic situations because it can potentially identify inadequate adherence before viral loads have begun to climb. However, their modest association with viral failure suggests that some non-adherent patients report that they are adherent. By contrast, pharmacy refill data give a longer term picture of adherence that is more strongly related to subsequent viral failure than self-reported adherence.

Advantages of pharmacy refill data include being inexpensive and immune to social desirability/recall bias or tampering [8,11]. Such data usually [8,21], although not always [22], have at least moderate correlations with virologic outcomes. Potential errors in adherence measure through pharmacy refill may occur if periods of undersupply are compensated for by oversupply [21]. Further, pharmacy refill records will not reflect patients obtaining medications through alternative sources, such as free samples, family or friends, or other pharmacies, which may occur in the most vulnerable patients [8]. These concerns are highlighted by studies that have described groups of patients who were highly adherent as measured by pharmacy refill data, but did not achieve or maintain viral suppression, and poorly adherent patients who maintained undetectable viral load [11,23]. Such problems are unlikely to have occurred in our study, because other sources of obtaining treatment are limited. Nonetheless, pharmacy refill data should be considered a measure of drug availability, or refill compliance, rather than a direct measure of consumption and, therefore, we cannot draw strong conclusions on levels of drug required for suppression. Further, medication is not always taken correctly as prescribed. The meaning of pharmacy data may differ between clinics with a “push” system (patients automatically get new medications when required) or those with a “pull” system (patients have to request medications when required).

Self-reported adherence, in context of viral load monitoring, is commonly used in both clinical care and research settings, because of its low respondent and staff burden, ease and speed of administration, and low cost [8,24]. The real-time nature of self-report questionnaires may prompt discussions with providers regarding reasons for poor adherence and potential solutions [8], which may explain the lower discriminatory power of self-report data compared with pharmacy data. A systematic review of 77 studies of self-reported adherence found it to be significantly correlated with viral load in 84% of recall periods [24]. A meta-analysis that included 65 studies found that the odds of viral failure were more than doubled in patients who self-reported inadequate or poor adherence compared with good adherence [25]. However, a common finding is that self-report data are susceptible to recall bias, inaccurate memory, and social desirability bias [24]. Collecting adherence data via self-report questionnaires may be difficult, or impossible, in cognitively impaired individuals [24].

Several studies have compared self-report to pharmacy refill, both among HIV-infected individuals and other disease conditions, such as chronic obstructive pulmonary disease medications [26]. Self-report has often performed poorly compared with pharmacy measures, particularly in settings such as intervention trials [26], and where there are interviewer-based measures of adherence. In these settings, there may be consequences to poor adherence, or increased pressure to overreport adherence. Depending on how the measurements are done and in what patient population, studies have found no relationship between self-report and pharmacy refill [27], or only poor correlations [26]. Comparisons between studies with self-report measures can be difficult, due to the lack of standardised instruments, including differences in the questions asked, the response format, and the recall period. For example, some questionnaires ask patients to recall the last 3 days and others ask patients to recall up to 28 days or more. The recommended recall period is 1 month [28]. Although, when we considered a recall period of 12 months in the VACS data, we observed a strong correlation between this and the pharmacy measure.

### 4.3. Strengths and Weaknesses

We investigated how adherence to ART is measured across cohorts from two continents providing routine clinical care in high-income settings. These data are representative of patients in usual clinical care and, so, it is likely that our results are generalisable to other high-income settings. Our analyses may be subject to a number of biases. We assumed that viral load measures should be done every 6 months: if a viral load measurement was not available within this time period, then we had to exclude the patient from analyses. Thus, patients included in analyses may have been more adherent to care than excluded patients. On the other hand, less adherent patients may have been monitored more closely and, therefore, be more likely to be included. Questionnaires were not routinely administered at regular time intervals and, therefore, it was not feasible to derive a 1-year self-report adherence measure without heavily reducing the sample size or making strong assumptions about adherence in the time between questionnaires. Association of adherence levels with viral failure may be different for newer ART regimens which have longer half-lives. 

## 5. Implications

Cohorts should consider carefully how best to collect and make use of adherence data for patient care within their healthcare setting. Pharmacy refill data require time and effort for their management, but provided a better tool, than self-report data, for predicting viral failure in our study. Harmonisation of pharmacy refill data management should be attempted in order to ensure that we are using the same tools. Without this, we need to recognise that differences in dispensing will influence refill data. Refill data are not as useful as self-report data in facilitating immediate interventions to change behaviour, which may prevent patients from experiencing subsequent viral failure. 

## Figures and Tables

**Table 1:** Baseline patient and cohort characteristics, *N* = 11,689.

		Pharmacy refills				Self-Report			
Characteristic	*N* (%)	Alberta	HOMER	VACS	HAVACS	ICONA	Washington	SHCS	UAB
Total, *N* (%)	1,1689	485 (4.2)	2241 (19.2)	4404 (37.8)	26 (0.2)	203 (1.7)	198 (1.7)	3607 (30.9)	525 (4.5)
Female, *N* (%)	1883 (16.1)	97 (20.0)	386 (17.2)	111 (2.5)	0 (0)	42 (20.7)	29 (14.7)	1114 (30.9)	104 (19.8)
Age (median, IQR)	41 (34–49)	38 (32–44)	40 (33–47)	46 (39–52)	43 (39–50)	37 (32–43)	37 (31–44)	37 (31–44)	37 (29–45)
Transmission risk group									
MSM	2667 (22.8)	235 (48.5)	494 (22.0)	66 (1.5)	11 (42.3)	67 (33.0)	100 (50.5)	1384 (38.4)	310 (59.1)
IDU	1824 (15.6)	73 (15.1)	796 (35.5)	574 (13.0)	6 (23.1)	41 (20.2)	52 (26.3)	263 (7.3)	19 (3.6)
Heterosexual	2291 (19.6)	167 (34.4)	164 (7.3)	7 (0.2)	2 (7.7)	85 (41.9)	0 (0)	1671 (46.3)	195 (37.1)
Blood	107 (0.9)	7 (1.4)	0 (0)	24 (0.5)	0 (0)	0 (0)	46 (23.2)	30 (0.8)	0 (0)
Other/unknown	4800 (41.1)	3 (0.6)	787 (35.1)	3733 (84.8)	7 (26.9)	10 (4.9)	0 (0)	259 (7.2)	1 (0.2)
Median (IQR) CD4 count (cells/mm^3^)	200 (80–320)	178 (69–281)	180 (80–300)	192 (61–333)	140 (54–280)	285 (178–375)	213 (72–271)	207 (104–315)	239 (62–358)
Median (IQR) viral load (log_10_ copies/mL)	4.9 (4.4–5.3)	4.8 (4.2–5.3)	5.0 (4.7–5.2)	4.9 (4.4–5.3)	4.8 (4.5–5.3)	4.8 (4.4–5.3)	5.1 (4.6–5.6)	4.9 (4.4–5.4)	4.9 (4.3-5.4)
AIDS at baseline	2711 (23.2)	121 (25.0)	405 (18.1)	1244 (28.3)	10 (38.5)	13 (6.4)	42 (21.2)	701 (19.4)	175 (33.3)
Mean (SD) % adherence,	88.7 (20.1)	89.5 (20.5)	85.2 (24.8)	80.8 (22.0)	80.3 (20.9)	97.4 (11.2)	94.9 (11.4)	99.0 (5.1)	94.1 (15.2)
Median (IQR) year of adherence measurement	2003 (2000–2006	2006 (2005–2007)	2001 (1998–2004)	2000 (1998–2002)	2002 (2001–2005)	2010 (2009–2011)	2009 (2008–2010)	2004 (2003–2007)	2009 (2008–2011)
Number of sites in cohorts *		1	500	125	1	32	1	7	1
Location (regional or national cohort?)		Regional	Regional	National	Regional	National	Regional	National	Regional
Is prescription filled at once or with refills?		Refills	Refills	Refills	Refills	Refills	Refills	Refills	Refills
Is refill drug supplied automatically without patient requesting it?		No	No	No	Yes	Yes	No	No	Yes if mail order drug

MSM: Men who have sex with men, IDU: Injection drug users. SD: standard deviation. IQR: Interquartile range. * July 2013.

**Table 2:** Viral suppression and levels of adherence across cohorts. Odds ratios (OR) and 95% CI for associations of adherence with viral failure.

	Pharmacy Refill Data				Self-Report Data			
Cohort	Alberta (Canada)	HOMER (Canada)	VACS (USA)	HAVACS (USA)	ICONA (Italy)	Washington (USA)	SHCS (Switzerland)	UAB (USA)
*N*	485	2241	4404	26	203	198	3607	525
% suppressed	89.3	73.7	63.0	61.5	95.6	95.0	91.7	86.9
Median (IQR) % adherence	100 (90–100)	100 (80–100)	89 (68–100)	85 (70–100)	100 (100–100)	99 (94–100)	100 (100–100)	99 (96–100)
Among those suppressed	100 (95–100)	100 (100–100)	94 (80–100)	85 (71–96)	100 (100–100)	99 (94–100)	100 (100–100)	99 (96–100)
Among those not suppressed	49 (27–89)	61 (33–88)	73 (52–92)	87 (70–100)	100 (95–100)	97 (90–100)	100 (98–100)	98 (91–100)
Proportion with ≥95% adherence	67%	62%	39%	38%	91%	74%	96%	81%
Proportion with 100% adherence	52%	60%	28%	27%	85%	36%	82%	37%
Adherence								
Lower	6.4	10.6	9.0	7.7	12.3	31.3	4.3	22.3
Medium	36.9	29.1	60.7	65.4	2.5	32.3	13.3	40.7
Higher	56.7	60.3	30.3	26.9	85.2	36.4	82.4	37.0
OR (95% CI) lower ^†^ vs. higher	80.4 (27.8,233)	85.2 (55.6,131)	12.3 (9.42,16.1)	0.75 (0.03,17.5)	3.8 (0.9,16.3)	1.2 (0.3,4.9)	4.4 (3.0,6.4)	2.0 (1.1,3.8)
OR (95% CI) medium ^†^ vs. higher	5.9 (2.5,14.1)	10.5 (8.1,13.6)	2.6 (2.2,3.0)	0.3 (0.1,1.9)	*	0.5 (0.1,3.1)	1.2 (0.8,1.7)	0.8 (0.4,1.5)
OR (95% CI) lower vs. medium	13.6 (5.7,32.3)	8.1 (5.4,12.1)	4.8 (3.7,6.1)	2.4 (0.1,46.4)	*	2.1 (0.4,12.1)	3.7 (2.3,6.0)	2.5 (1.3,4.7)
Overall ORs								
OR (95% CI) lower ^†^ vs. higher	26.13 (21.05, 32.42)				3.14 (2.36, 4.19)			
OR (95% CI) medium ^†^ vs. higher	4.41 (3.88, 5.01)				1.15 (0.86, 1.53)			
OR (95% CI) lower ^†^ vs. medium	5.92 (4.86, 7.23)				2.74 (1.91, 3.95)			
Adjusted ORs								
OR (95% CI) lower ^†^ vs. higher	23.04 (18.44, 28.78)				3.19 (2.31, 4.40)			
OR (95% CI) medium ^†^ vs. higher	3.84 (3.36, 4.39)				1.08 (0.80, 1.46)			
OR (95% CI) lower ^†^ vs. medium	5.73 (4.67, 7.04)				3.13 (2.13, 4.59)			

* Predicts suppression perfectly; ^†^ For pharmacy data lower is <45% adherent, medium 45–99%, higher 100%. For self-report data lower is ≤95%, medium 96–99%, higher 100%.

## Supplementary Materials

The following are available online at http://www.mdpi.com/2077-0383/7/10/328/s1, Web Table 1: Odds ratios (95% CI) for failure according to adherence and age, Web Table 2: Odds ratios (95% CI) for failure using the cut-offs of ≤500 cp/mL and ≤50 cp/mL, Web Table 3: Odds ratios (95% CI) for adherence on viral failure using the cut-off of ≤ 500 cp/mL and restricting to data from 2009 onwards, *N* = 885, Web Table 4: Odds ratios (95% CI) for adherence on viral failure using the cut-off of ≤500 cp/mL and restricting to patients still on their baseline regimen, *N* = 9740, Web Table 5: Description of pharmacy adherence measures when calculated over 3, 6 and 12 months, Web Table 6: Summary of pharmacy adherence calculation according to self-reported adherence, Web Table 7: Odds Ratio of adherence measure with viral failure, *N* = 331.
