# Supplementary material for: Identifying Risk of Viral Failure in Treated HIV-Infected Patients Using Different Measures of Adherence: The Antiretroviral Therapy Cohort Collaboration
Source: J Clin Med. 2018 Oct 5;7(10):328. doi: 10.3390/jcm7100328 (PMC6209956; doi:10.3390/jcm7100328)
Supplement: Supplementary file 1 [file jcm-07-00328-s001.pdf]

Web Table 1: Odds ratios (95% CI) for failure according to adherence and age.

Covariate	*N*	Age 16-29	Age 30-39	Age 40-49	Age 50+
Cohorts with pharmacy refill data	7156				
Adherence					
Low	667	52.5 (17.7,156.0)	32.9 (19.3,56.3)	23.3 (15.3,35.5)	15.5 (9.3,25.9)
Medium	3519	6.3 (3.7,10.8)	5.2 (4.0,6.7)	4.3 (3.5,5.3)	3.6 (2.8,4.6)
High*	2970	1	1	1	1
P for interaction=0.0612					
Cohorts with self-report data	4533				
Adherence					
Low	359	3.6 (2.0,6.4)	2.4 (1.5,3.9)	3.2 (1.7,5.9)	4.9 (2.3,10.5)
Medium	764	1.5 (0.9,2.6)	1.2 (0.7,1.8)	0.8 (0.4,1.6)	1.2 (0.5,2.6)
High*	3410	1	1	1	1
P for interaction=0.4465					

* Reference category.

Web Table 2: Odds ratios (95% CI) for failure using the cut-offs of ≤500 cp/mL and ≤50 cp/mL.

		≤500 cp/ml, *N* = 11,689			≤50 cp/mL, *N* = 8272	
		Crude	Adjusted*		Crude	Adjusted*
Covariate	N	OR (95% CI)	OR (95% CI)		OR (95% CI)	OR (95% CI)
Adherence						
Low	1026	12.9 (11.1,15.0)	12.4 (10.5,14.7)	586	7.8 (6.4, 9.3)	8.3 (6.7,10.3)
Medium	4283	4.6 (4.2,5.1)	2.9 (2.6,3.3)	2549	3.2 (2.8,3.5)	2.3 (2.0,2.6)
High	6380	1	1	5137	1	1

*adjusted for: cohort, age, gender, CD4 and viral load at ART start, AIDS at ART start, and transmission risk group.

Web Table 3: Odds ratios (95% CI) for adherence on viral failure using the cut-off of ≤500 cp/mL and restricting to data from 2009 onwards, $N = 885$.

		**Pharmacy data***			**Self-report data**	
		Crude	Adjusted**		Crude	Adjusted*
Covariate	*N*	OR (95% CI)	OR (95% CI)	*N*	OR (95% CI)	OR (95% CI)
Adherence						
Low	0	N/A	N/A	153	3.1 (1.7,5.6)	3.0 (1.5,5.9)
Medium	5	N/A	N/A	224	1.4 (0.8,2.7)	1.1 (0.6,2.3)
High	2	N/A	N/A	501	1	1

*There is no variability in viral failure for this subset of data and therefore logistic regression cannot be run. ** adjusted for: cohort, age, gender, CD4 and viral load at ART start, AIDS at ART start, and transmission risk group

Web Table 4: Odds ratios (95% CI) for adherence on viral failure using the cut-off of ≤500 cp/mL and restricting to patients still on their baseline regimen, $N = 9740$.

		**Pharmacy data**			**Self-report data**	
		Crude	Adjusted*		Crude	Adjusted*
Covariate	*N*	OR (95% CI)	OR (95% CI)	*N*	OR (95% CI)	OR (95% CI)
Adherence						
Low	656	26.0 (20.9, 32.4)	22.9 (18.3, 28.7)	187	3.0 (2.0,4.6)	3.7 (2.3,5.9)
Medium	3430	4.4 (3.9, 5.1)	3.9 (3.4, 4.4)	429	1.0 (0.6,1.5)	1.0 (0.6,1.6)
High	2873	1	1	2165	1	1

*adjusted for: cohort, age, gender, CD4 and viral load at ART start, AIDS at ART start, and transmission risk group

Web Table 5: Description of pharmacy adherence measures when calculated over 3, 6 and 12 months.

	**3 months**	**6 months**	**12 months**
Alberta			
N	490	473	485
% suppressed	90%	90%	89%
Median percentage adherence	100	99	100
Among those suppressed	100	100	100
Among those not suppressed	72	64	49
Proportion with ≥95% adherence	71%	65%	67%
Proportion with 100% adherence	51%	47%	52%
AUROC	0.7328	0.8116	0.8577
HOMER			
N	2310	2246	2241
% suppressed	77%	76%	74%
Median percentage adherence	100	100	100
Among those suppressed	100	100	100
Among those not suppressed	86	67	61
Proportion with ≥95% adherence	79%	65%	62%
Proportion with 100% adherence	78%	64%	60%
AUROC	0.7346	0.7918	0.8548
VACS			
N	5175	5055	4404
% suppressed	65%	65%	63%
Median percentage adherence	99	98	89
Among those suppressed	100	100	94
Among those not suppressed	90	81	73
Proportion with ≥95% adherence	64%	57%	39%
Proportion with 100% adherence	49%	47%	28%
AUROC	0.6544	0.6882	0.7157
HAVACS			
N	49	51	26
% suppressed	57%	47%	62%
Median percentage adherence	97	90	85
Among those suppressed	84	87	85
Among those not suppressed	97	90	87
Proportion with ≥95% adherence	51%	41%	38%
Proportion with 100% adherence	39%	31%	27%
AUROC	0.4294	0.4931	0.4531

Web Table 6: Summary of pharmacy adherence calculation according to self-reported adherence.

**Self-reported adherence in the last 12 months**	**Pharmacy adherence in the same 12 month period**	
	N	Median (IQR)
Never	9	31.5 (17.3-81.1)
Some	13	55.6 (42.9-97.3)
About half	5	69.0 (32.9-81.1)
Most of the time	135	90.4 (64.9-100)
All of the time	186	100 (87.9-100)
Total	348	98.1 (72.1-100)

Web Table 7: Odds Ratio of adherence measure with viral failure, *N* = 331.

**Adherence measure**	***N***	**OR for viral failure (95% CI)**	**AUROC using non-dichotomised measure**
12-month self-report			0.619
Never	9	6.9 (1.4,34.4)	
Some	13	10.9 (2.3,50.8)	
About half	3	4.0 (0.4,44.6)	
Most of the time	127	1.8 (1.2,2.9)	
All of the time	179	1	
12-month pharmacy adherence categories			0.628
Low	33	4.9 (2.1,11.1)	
Medium	135	1.4 (0.9,2.2)	
High	163	1	
